# Adjudicating Between Local and Global Architectures of Predictive Processing in the Subcortical Auditory Pathway

**DOI:** 10.3389/fncir.2021.644743

**Published:** 2021-03-12

**Authors:** Alejandro Tabas, Katharina von Kriegstein

**Affiliations:** ^1^Chair of Cognitive and Clinical Neuroscience, Faculty of Psychology, Technische Universität Dresden, Dresden, Germany; ^2^Max Planck Institute for Human Cognitive and Brain Sciences, Leipzig, Germany

**Keywords:** predictive coding, medial geniculate body, inferior colliculus, abstract processing, sensory coding, auditory processing, subcortical sensory pathway

## Abstract

Predictive processing, a leading theoretical framework for sensory processing, suggests that the brain constantly generates predictions on the sensory world and that perception emerges from the comparison between these predictions and the actual sensory input. This requires two distinct neural elements: generative units, which encode the model of the sensory world; and prediction error units, which compare these predictions against the sensory input. Although predictive processing is generally portrayed as a theory of cerebral cortex function, animal and human studies over the last decade have robustly shown the ubiquitous presence of prediction error responses in several nuclei of the auditory, somatosensory, and visual subcortical pathways. In the auditory modality, prediction error is typically elicited using so-called oddball paradigms, where sequences of repeated pure tones with the same pitch are at unpredictable intervals substituted by a tone of deviant frequency. Repeated sounds become predictable promptly and elicit decreasing prediction error; deviant tones break these predictions and elicit large prediction errors. The simplicity of the rules inducing predictability make oddball paradigms agnostic about the origin of the predictions. Here, we introduce two possible models of the organizational topology of the predictive processing auditory network: (1) the global view, that assumes that predictions on the sensory input are generated at high-order levels of the cerebral cortex and transmitted in a cascade of generative models to the subcortical sensory pathways; and (2) the local view, that assumes that independent local models, computed using local information, are used to perform predictions at each processing stage. In the global view information encoding is optimized globally but biases sensory representations along the entire brain according to the subjective views of the observer. The local view results in a diminished coding efficiency, but guarantees in return a robust encoding of the features of sensory input at each processing stage. Although most experimental results to-date are ambiguous in this respect, recent evidence favors the global model.

## 1. Introduction

The massive bundle of corticofugal fibers stemming from auditory cortex and targeting nuclei of the subcortical auditory pathway (Winer, 1984, 2005b; Schofield, 2011) have posed a puzzling problem to the auditory neuroscience community for decades (Syka et al., 1988; Winer, 2005a; Robinson and McAlpine, 2009; He and Yu, 2010). Sensory processing is classically understood as a bottom up problem, where increasingly complex features are read-out in a hierarchical constructive manner (Epstein, 1993; Martin, 1994; DeCharms and Zador, 2000). But then, what is the corticofugal system good for, and why is it that massive?

One possibility is that sensory processing is not a purely bottom-up process, but that top-down information is used proactively to encode sensory input (Mumford, 1992; Rao and Ballard, 1999; Friston, 2003, 2005). This is the thesis of the predictive processing framework (PPF) (Heeger, 2017; Spratling, 2017; Keller and Mrsic-Flogel, 2018; Walsh et al., 2020): that higher-level regions of the cerebral cortex keep and update a model of the sensory world that is used to predict, in a generative manner, the sensory input at lower-level regions of the cerebral cortex; and that neurons at those lower-level regions encode *prediction error*: the difference between the predictions and the actual input. Prediction error is further conveyed to the higher-level representation and used there to adjust the generative model. Extending this role to the corticofugal system between cerebral cortex and subcortical sensory pathway nuclei suggests that predictions drawn by generative models in cerebral cortex are conveyed to subcortical sensory neurons that encode prediction error (Von Kriegstein et al., 2008; Diaz et al., 2012; Malmierca et al., 2015). Many authors have argued that the PPF might underlay cognitive processes beyond perception including (e.g.,): vocalization in humans (Okada et al., 2018) and birds (Yildiz and Kiebel, 2011), learning in cognitive development (Nagai, 2019), episodic memory (Barron et al., 2020), abstract cognition and reasoning (Spratling, 2016), inculturation (Fabry, 2018), and even the emergence of faith (Andersen, 2019). Here we focus on sensory processing and, in particular, on auditory perception.

Over the last decade the auditory neuroscience community has robustly shown the predominance of neurons encoding prediction error neurons in subcortical sensory pathway nuclei (Anderson et al., 2009; Malmierca et al., 2009, 2014, 2019; Grimm et al., 2016; Parras et al., 2017; Carbajal and Malmierca, 2018). Although these results are often taken as proof that the corticofugal system is indeed transmitting predictions, most experimental paradigms control predictability using simple rules that can be readily encoded at the same processing stage as the prediction error (Eytan et al., 2003; Mill et al., 2011; Wang et al., 2014; May et al., 2015); i.e., without needing a top-down system. We will call this the ‘local model’ of predictive coding in the following. Conversely, we refer to predictive coding as a ‘global model’ if a generative model at higher-levels of the processing hierarchy generates predictions for the lower levels. The distinction between local and global models of predictive coding is important for understanding the function of the corticofugal pathway. It is also important for the understanding of the nature of sensory processing: if predictions are computed at higher stages of the processing hierarchy and transmitted downwards, that would mean that the auditory system can only make sense of stimuli that are conceivable at these higher-level representations.

Predictability plays an important role in sensory processing: there are many benefits of predictability on behavioral performance in the neurotypical brain (e.g., Davis and Johnsrude, 2007; Jaramillo and Zador, 2011; Sohoglu and Davis, 2016; Mazzucato et al., 2019). If using such predictability for understanding the world is not possible, this likely results in dysfunction. Deficits in the predictive elements of the PPF have already been suggested to explain a number of symptoms in neuropsychiatric conditions, including in schizophrenia (Horga et al., 2014; Sterzer et al., 2018, 2019), autism-spectrum disorders (van de Cruys et al., 2014; van Schalkwyk et al., 2017), attention deficit and hyperactivity (Gonzalez-Gadea et al., 2015), and mood and eating disorders (Frank et al., 2016; Clark et al., 2018). Deficits in the predictive elements of the PPF have also been directly linked to dysfunction of cortico-thalamic pathways and sensory auditory thalamus in developmental dyslexia (Diaz et al., 2012; Müller-Axt et al., 2017; Tschentscher et al., 2019). Understanding the computational mechanism for encoding predictability and the role of the corticofugal system in predictive sensory processing is a necessary prerequisite for a mechanistic understanding of these disorders.

In the following, we review the existing literature on predictive processing in the auditory sensory system with a special focus on the potential role of the corticofugal pathway. We focus on audition because it is the modality where subcortical predictive processing has been explored the most in the last decade (Nelken and Ulanovsky, 2007; Garrido et al., 2009; Grimm et al., 2011; Escera and Malmierca, 2014; Malmierca et al., 2015; Heilbron and Chait, 2017; Carbajal and Malmierca, 2018).

## 2. Global and Local Models of the Predictive Processing Framework

**Figure 1:**
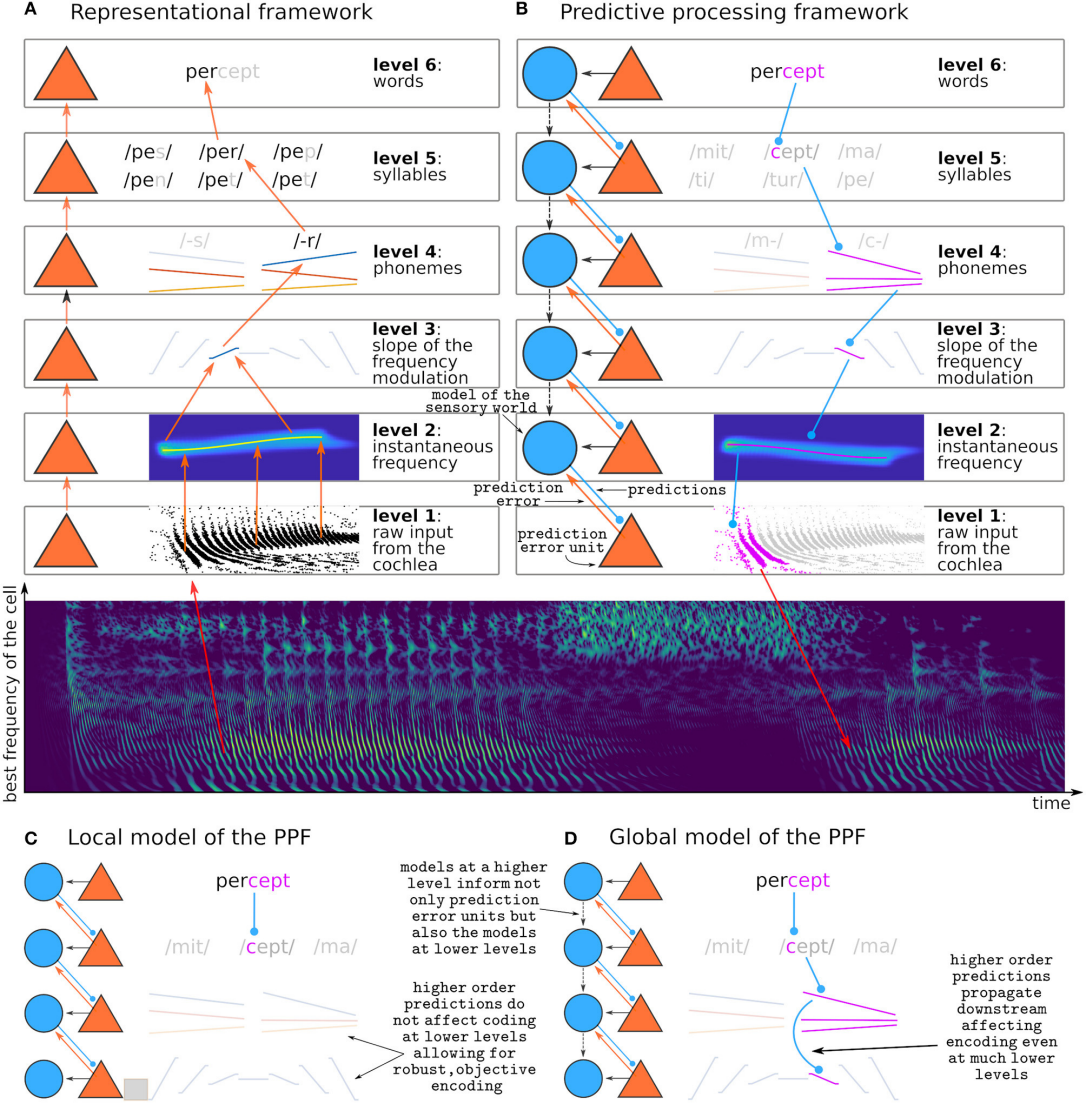
Representational and predictive processing frameworks. **(A)** Schematic view of a possible implementation of the representational framework during the decoding of the syllable */per-/*. **(B)** Schematic view of a possible implementation of the predictive processing framework while performing predictions on the incoming syllable */-cept/*. Purple features are predictions. The middle panel depicts a simulation of the neural activity in the auditory nerve across the tonotopic axis (*y*-axis) elicited by the spoken word *percept*. **(C)** Example implementation of the local model: whereas predictions at the word level are used to encode prediction error at the immediately lower level as formulated by the PPF, predictions are not used to calculate the predictive model of the lower stages, and thus the remaining levels depend on bottom-up information only. **(D)** Example implementation of the global model: predictions at the word level propagate downwards affecting the predictive model of all stages (dashed descending arrows), so that prediction error at the lowest level is encoded with respect to a model that is ultimately defined in the highest level. Intermediate implementations where interactions between the models exist up to a certain level only are also possible but not displayed here. Note that **(B)** depicts the global model.

A longstanding question on sensory processing is whether perception is purely exploratory or rather a process of inference (Von Helmholtz, 1867; Atick, 1992; Bejjanki et al., 2011; Lochmann and Deneve, 2011; Purves et al., 2015; de Lange et al., 2018). In the exploratory view, observers passively receive information from their senses and construct a representation of their sensory surrounds based on a lump of perceptual objects (Epstein, 1993; Martin, 1994; Quiroga et al., 2005; Chechik et al., 2006; Wood et al., 2019). The exploratory view is implemented by the so-called *representational framework* of sensory processing (Epstein, 1993; DeCharms and Zador, 2000; DiCarlo et al., 2012). The representational framework sees perception as a constructive process carried out by a cascade of *feature detectors*: neurons that analyse neural activity at the immediately lower hierarchical stage and respond selectively to certain activation patterns (Figure 1A). For instance, a neuron that responds selectively to the word *percept* integrates inputs from neurons encoding the syllables *per* and *cept*; the neuron encoding *per* receives inputs from neurons encoding *p-, e*, and *-r*; and, if the word is decoded from the auditory modality, the neuron encoding *p-* receives inputs from the neurons encoding each of the formant transitions (frequency-modulated sweeps) that characterize the consonant.

The PPF (Heeger, 2017; Spratling, 2017; Keller and Mrsic-Flogel, 2018; Walsh et al., 2020) presents the same hierarchical organization as the representational framework, but the feature detectors are used for inference rather than exploration. In the PPF, a feature detector needs two ingredients: a *(generative) model* that builds hypotheses about the sensory world, and a *prediction error* unit that tests these hypotheses against the actual sensory input. The PPF is intimately linked with Bayesian inference (Friston, 2005; Kiebel et al., 2008), where the posterior conclusions drawn from the data are amplifications or reductions of a prior belief. This means that according to the PPF we are more likely to perceive what we expect. In an extreme interpretation of the framework, it implies that if we do not have an implicit prior belief that a perceptual object might exist, we cannot perceive its existence at all. Today, evidence from psychophysics (de Lange et al., 2018), human neuroimaging (Siman-Tov et al., 2019; Walsh et al., 2020), animal neurophysiology (Bendixen et al., 2012; Carbajal and Malmierca, 2018; Pakan et al., 2018), and theoretical neuroscience (Brenner et al., 2000; Fairhall et al., 2001; Huang and Rao, 2011; Badcock et al., 2019), converges in the idea that predictions on the sensory world are constantly used to encode sensory input.

To-date there are at least three different algorithms describing how the PPF could be implemented in the brain (for a review, see Spratling, 2017). All of them hypothesize the existence of two kinds of sensory neurons: those that encode the generative model, and those that encode prediction error. A neuron encoding the generative model at a certain stage of the processing hierarchy *k* receives inputs from its associated prediction error units, that signal if the model is correct or incorrect. It also receives input from generative models at higher stages *l* > *k*, that guide the generation of predictions at level *k*. A prediction error unit at stage *k* compares the predictions of its associated generative model at stage *k*+1 with the sensory inputs incoming from the immediately lower processing stage (Figure 1B).

Although the PPF was first formulated as a theory on sensory processing in the cerebral cortex (Rao and Ballard, 1999; Friston, 2005; Shipp, 2016), the existence of potential prediction error units in the subcortical auditory pathway has been reported widely during the last decade (Anderson et al., 2009; Malmierca et al., 2009, 2014, 2019; Grimm et al., 2016; Parras et al., 2017; Carbajal and Malmierca, 2018). Whether this prediction error is, as proposed by the PPF, a signature of active inference, is still unclear (Carbajal and Malmierca, 2018). If that was the case, prediction error in subcortical sensory structures should signal error with respect to global hypotheses of the sensory world. This means that, if after hearing *per-* we expect the word to be completed with a *-cept* (Figure 1B), an auditory signal breaking such prediction (like, for instance, *-meable*) should elicit prediction errors in those neurons encoding the syllable *-cept*, but also errors on the neurons encoding the *-c-* and its corresponding spectrotemporal properties such as formant transitions. This schema assumes that predictions are transmitted downwards through an inverse hierarchical structure (Figure 1D). We call this the *global model*, because it assumes that predictions at the highest stage in the processing hierarchy are used to inform generative models globally across the brain (Kiebel et al., 2008; Malmierca et al., 2015; Siman-Tov et al., 2019; Casado-Román et al., 2020).

An alternative possibility is that predictions and its associated errors are computed locally (Eytan et al., 2003; Mill et al., 2011; Wang et al., 2014; May et al., 2015). We call this scenario the *local model*. Predictions at each stage are performed accordingly to the level of abstraction of the local representation and its local time constant of integration. In this scenario, violations to the prediction *-cept* would elicit prediction error in the populations encoding the syllable *-cept*, but not in the populations encoding the formant transitions of the syllable *-c-* (Figure 1C). Although not strictly adherent to the principles of the PPF, this local strategy presents its own advantages. First, it still optimizes the neural code by encoding only those parts of the stimulus that are not predictable. Second, it keeps robust representations of the stimuli that are independent of each other across stages of the processing hierarchy - this has the advantage that it could be used to simultaneously test multiple hypotheses. Third, it does not require a constant top-down transmission of expectations.

## 3. Prediction Error Responses Are Ubiquitous in the Auditory Thalamus and Midbrain

Prediction error responses in the mammal subcortical auditory pathway (Malmierca et al., 2015; Parras et al., 2017) have been mostly investigated through stimulus-specific adaptation (SSA). SSA is a phenomenon where individual sensory neurons adapt to specific stimulus properties (Ulanovsky et al., 2003, 2004). SSA is typically shown in passive listening conditions (and often in anesthetized animals) using some variation of the classical oddball paradigm: sequences of several repetitions of a standard tone that are interrupted by rarely occurring deviants. Deviants typically differ from the standards only in the tone frequency. In oddball sequences, pure tones are separated by fixed inter-stimulus-intervals (ISI), so that the onset of the next tone in the sequence is always predictable. By repetition of the standard tones, oddball paradigms induce predictions on the frequency of the next tone. The experimenter can control the amount of prediction error elicited by the deviants with two variables: the frequency difference between deviant and standard (which controls the amount of error of the prediction with respect to the actual sensory input) and the probability of occurrence of the deviant (which controls how certain the model is about the prediction that the next stimulus will be a standard). SSA to frequency deviants has been consistently found in the auditory thalamus (medial geniculate body, MGB) (Anderson et al., 2009; Antunes et al., 2010; Richardson et al., 2013; Duque et al., 2014; Parras et al., 2017) and midbrain (inferior colliculus, IC) (Malmierca et al., 2009; Zhao et al., 2011; Duque et al., 2012; Pérez-González et al., 2012; Ayala et al., 2013, 2015, 2016; Ayala and Malmierca, 2015, 2018; Duque and Malmierca, 2015; Parras et al., 2017; Valdés-Baizabal et al., 2017, 2020) of non-human mammals, as well as in the human IC and MGB (Cacciaglia et al., 2015; Tabas et al., 2020). Several studies have failed to detect any SSA in neurons or populations of the first stage of the auditory subcortical pathway: the auditory brainstem (cochlear nucleus, CN) (Duque et al., 2012, 2018; Ayala et al., 2013, 2015; Parras et al., 2017). Although SSA has been mostly investigated using frequency deviants, similar adaptation dynamics have been demonstrated to amplitude modulation deviants (Gao et al., 2014) and, in bats, to frequency modulation deviants (Thomas et al., 2012). However, there seems to be no SSA to loudness deviants (Duque et al., 2016). The SSA magnitude is typically measured with the SSA index, a ratio that compares the neuronal responses to a tone when used as a standard with the responses to the same tone when used as a deviant.

Although positive SSA indices are often taken as an indication that the neuron encodes prediction error (i.e., surprise to the violation of a prediction), positive SSA indices could also result from simple repetition suppression to the standard (Taaseh et al., 2011; Parras et al., 2017; Carbajal and Malmierca, 2018). Parras et al. developed a novel approach to disentangle repetition suppression from prediction error by comparing the responses to deviants in classical oddball sequences with the responses to the same sounds when embedded in control sequences that contain varying non-predictable stimuli. They argued that, if the responses to deviants encoded prediction error, these responses should be stronger when a precise prediction on the incoming stimuli is available (as in oddball sequences) than when predictions are broad (as in the control sequences, where all control stimuli have the same likelihood of occurrence). They termed the difference of the responses to the deviant tone in oddball and control sequences the *index of prediction error* (iPE), and demonstrated that neurons showing SSA do typically show positive iPEs. However, iPE>0 is not a sufficient indication of prediction error because, as modeling studies have shown (Eytan et al., 2003; Mill et al., 2011, 2012), positive iPEs can also arise due to simple repetition suppression due to suppressed inhibition. In any case, it is useful to consider SSA as an aggregation of two separate phenomena: the suppression of the responses to the standards, and the recovery of the responses to the deviant.

The IC, MGB, and also auditory cortex are typically subdivided in primary and secondary subdivisions. The bulk of primary subdivisions, consisting of the entire CN, the central nucleus of the IC (cIC), and the ventral section of the MGB (vMGB), constitute the so-called *lemniscal* pathway, characterized by narrow frequency tuning bands and faithful encoding of the stimulus properties (Lee and Sherman, 2011).

The bulk of secondary subdivisions, consisting of the cortex of the IC (xIC), and the medial (mMGB) and dorsal (dMGB) sections of the MGB, constitute the *non-lemniscal* pathway, characterized by wider or absent frequency tuning and stronger corticofugal projections (Lee and Sherman, 2011). While the primary or lemniscal subdivision is attributed with the task of transmitting sensory information directly to the cerebral cortex, the secondary or non-lemniscal subvidision is thought to play a secondary role (Lee and Sherman, 2011). If the PPF is the main mechanism for sensory processing, it should govern sensory coding in the lemniscal pathway.

Animal studies seem to converge in that SSA is more prevalent (i.e., present in a larger fraction of neurons) and stronger (i.e., showing larger SSA indices) in non-lemniscal sections of IC and MGB (Anderson et al., 2009; Ayala et al., 2016; Parras et al., 2017). SSA neurons in the non-lemniscal pathways also show larger iPEs than their lemniscal counterparts. This finding is, however, not backed by studies in humans, which found no topological organization of SSA across IC or MGB (Cacciaglia et al., 2015; Tabas et al., 2020), or even reported comparable SSA indices in lemniscal and non-lemniscal MGB (Tabas et al., 2020).

SSA is elicited in IC and MGB in both, awake and anesthetized animals (Richardson et al., 2013; Duque and Malmierca, 2015; Parras et al., 2017), and under passive and active listening in humans (Cacciaglia et al., 2015; Tabas et al., 2020). One study reported higher SSA indices under anesthesia due to a global reduction of spontaneous activity (Duque and Malmierca, 2015); another study reported generally higher iPEs in the awake condition (Parras et al., 2017). Therefore, although SSA might be modulated by awareness, it is also present in states of reduced consciousness. This is fully in line with the principles of the PPF, where inference on the sensory world is computed autonomously as a coding strategy, rather than as a conscious inference effort.

In IC and MGB, the SSA index always increases with increasing frequency difference between deviant and standard, with decreasing ISI, and with decreasing probability of occurrence of the deviant. This phenomenology seems to indicate that neurons showing SSA do encode prediction error with respect to the hypothesis that the next tone will be a standard, and that this error is larger when there is a precise hypothesis than when there is none. Whether this model is computed locally (in the IC or the MGB) or globally and projected across the hierarchy (see Figures 1C,D) is still unclear. Early studies interpreted the fact that SSA is more prominent in non-lemniscal subdivisions of the rodent auditory pathway as evidence of global computation (Malmierca et al., 2015; Ayala et al., 2016). Later, evidence that both SSA indices and iPE increase along the rodent ascending auditory pathway led to the interpretation that prediction error is also computed locally at each stage (Parras et al., 2017; Carbajal and Malmierca, 2018). Functional MRI (fMRI) studies in humans also indicated that IC and MGB showed stronger responses to sounds that broke the predictions than to sounds for which predictions were not available. They did, however, not find that these effects were more prominent in the non-lemniscal subdivisions (Cacciaglia et al., 2015; Tabas et al., 2020).

## 4. Encoding Fidelity in the Auditory Brainstem Is Enhanced by Repetition and Predictability

Electroencephalographic (EEG) methods present a much higher temporal resolution than fMRI (Buxton, 2009), allowing to measure directly the responses to each individual tone in the sequence. However, fine temporal resolution is offered at the expense of spatial precision: triangulating the origin of the evoked potentials in the brain is generally an ill-posed problem. This difficulty makes measuring responses from subcortical nuclei, particularly because they are located centrally in the brain, especially challenging (Boston and Moller, 1985; Coffey et al., 2019).

To-date, the only non-invasive measurements of subcortical auditory evoked activity are the auditory brainstem response (ABR) and the frequency-following response (FFR). The ABR (Jewett et al., 1970; Parkkonen et al., 2009) consist of a series of short transient auditory evoked potentials peaking within 8 ms after tone onset with sources ranging from the auditory nerve up to the MGB. Human ABRs do not seem to show SSA to broadband spectrum deviants (Slabu et al., 2010) nor loudness deviants (Althen et al., 2011).

The FFR (Gerken et al., 1975; Boston and Moller, 1985; Chandrasekaran and Kraus, 2010) is a component of the auditory evoked fields that is synchronized to the acoustical signal. Although the FFR partially stems from sources in cerebral cortex (Coffey et al., 2016), most generators seem to be subcortical (Bidelman, 2018; Coffey et al., 2019), especially when it is synchronized to stimulus frequencies above the cortical limit for phase-locking (estimated to be between 50 and 250 Hz).

Two studies have shown SSA of the absolute power of the FFR in a neighborhood of the frequencies characterizing the stimuli (Shiga et al., 2015; Alho et al., 2019). However, the entrainment of the FFR to the stimulus waveform seems to follow the exact opposite trend than SSA: an increased entrainment to standards as compared to deviants (Chandrasekaran et al., 2009; Strait et al., 2011; Slabu et al., 2012; Skoe et al., 2014; Lau et al., 2017; Font-Alaminos et al., 2020). We call this phenomenology *repetition entrainment enhancement*. The repetition entrainment enhancement of the FFR seems independent of stimulus class: it has been shown for syllables (Chandrasekaran et al., 2009; Strait et al., 2011; Slabu et al., 2012; Gorina-Careta et al., 2016; Lau et al., 2017; Alho et al., 2019), amplitude modulated tones (Shiga et al., 2015), pitch contours in Mandarin syllables (Skoe et al., 2014), and pure tones (Font-Alaminos et al., 2020). One of these studies showed that the repetition entrainment enhancement is present even when the onset of the sounds is not predictable (i.e., with jittered ISIs) (Slabu et al., 2012), although predictable onsets do result in lower FFR power and higher FFR entrainment (Gorina-Careta et al., 2016). Moreover, two studies showed that the magnitude of the repetition entrainment enhancement correlates with the ability of the subjects to recognize speech in noise (Chandrasekaran et al., 2009; Strait et al., 2011). The fact that the FFR adapts its properties to the stimulation history is contrary to the predictions of a representational framework. The FFR repetition entrainment enhancement is, however, also not straightforward to interpret within the PPF. If the FFR represented prediction error, we would expect a gradual decay of the signal (and with it its SNR and quality of the entrainment) with each repetition of a standard. Another possibility is that the FFR encodes the generative model of the sensory input, which becomes more and more precise with each repetition. However, if that was the case the FFR would always represent expectations, which means that we would expect an FFR tuned to the standard during the presentation of a deviant. It is possible that the generative model corrects itself quickly after detecting that the stimulus is not a standard, which would result in a reduction of the FFR entrainment to the deviant. If that was the case the entrainment to the deviants would be much weaker than to the first standards in the sequence; however, the literature reports that the deviant and first standard elicit the same FFR entrainment (Font-Alaminos et al., 2020). A last possibility is that the FFR has contributions from both, prediction error and generative model units, and that the balance between the contribution of one and the other results in the observed phenomenology.

## 5. Mixed Evidence on the Global Model Based on Deactivation of High-Order Processing Structures

Studies reviewed so far have established that sensory processing in the auditory pathway cannot be explained by a representational framework. The studies suggested that computation of expectations and prediction error is common to many mammals, and that it occurs even during sleep and anesthesia. However, all these studies use paradigms that have as a core feature repetition to induce predictions on the sensory input. This means that prediction error is computed with respect to a model of the sensory world that could have been generated locally, at the level of the IC and MGB, or globally, at a higher level of the processing hierarchy. Thus, results reviewed so far are ambivalent with respect to the actual organization of the PPF and are compatible with both, the local and global models. The next sections of this review focus on studies that tried to disentangle between these two possibilities.

Neural populations encoding higher levels of abstraction are thought to be encoded at successively higher stages in the processing hierarchy (Kiebel et al., 2008). This hierarchical organization is exquisitely presented in the auditory system, where the CN encodes a faithful representation of raw sensory input (Rhode and Smith, 1986), the IC and MGB encode intermediate features like formant transitions (Kuo and Wu, 2012), and auditory cortex encodes the identity of sounds as complex auditory objects (Chechik and Nelken, 2012). One way to test whether expectations are computed globally and transmitted downwards through the auditory hierarchy is to study whether SSA in IC and MGB depends on the cerebral cortex.

Three animal studies have compared SSA in subcortical sensory pathway nuclei before and during reversible deactivation of the ipsilateral auditory cortex. Two of the studies used a cryoloop to temporarily deactivate rat's auditory cortex, and measured SSA in neurons of the IC (Anderson and Malmierca, 2013) and MGB (Antunes and Malmierca, 2011). Both studies reported that SSA in single neurons was affected by deactivation of the cerebral cortex. The overall averaged amount of SSA in IC and MGB did, however, not significantly change during deactivation. The authors concluded that although cerebral cortex may modulate subcortical SSA, it does not generate it. This means that SSA cannot be solely driven by the global model of the PPF. In contrast, a third study (Bauerle et al., 2011) used muscimol to deactivate auditory cortex and measured SSA in neurons of vMGB (i.e., in the lemniscal section) in gerbils. The authors found that SSA was completely abolished after muscimol application, concluding that SSA indeed depends on cerebral cortex function, supporting the global model.

Divergences between the three studies could be caused by: (1) different deactivation methods, (2) different species, or (3) differences between the lemniscal and non-lemniscal pathways. Whereas deactivation by cryoloop allowed the investigators to show recovered responses after cortical inactivation, the longer recovery periods and possible diffusion of muscimol (Lomber, 1999) prevented Bauerle et al. from recording post-inactivation responses. Thus, Bauerle et al. could not completely rule out that the abolition of SSA after drug administration was due to irreversible damages induced during the application of the drug or diffusion of the drug into thalamus (Bauerle et al., 2011). Although the authors claim that side effects of muscimol were unlikely, reproduction of the results are needed to confirm that deactivation of auditory cortex abolishes SSA in vMGB.

Although the studies using the cryoloop (Antunes and Malmierca, 2011; Anderson and Malmierca, 2013) do not report whether neurons belong to the lemniscal or non-lemniscal subdivisions of the IC and MGB, the relatively elevated SSA indices [*SSAi* > 0.18 in IC (Anderson and Malmierca, 2013) and average *SSAi* ~ 0.31 in MGB (Antunes and Malmierca, 2011)] indicate that most recorded neurons in the cryoloop experiments belonged to the non-lemniscal subdivisions. In comparison, SSA indices from the vMGB in Bauerle et al. (2011) were around *SSAi* = 0.07, even though they used shorter ISIs and higher intensity levels than the cryoloop studies, which potentially elicits higher levels of SSA. One possibility is that the cerebral cortex triggers SSA only in the lemniscal pathway. This would be surprising, given that most corticofugal fibers target neurons in the non-lemniscal subdivisions of the IC and MGB (Lee and Sherman, 2011). Another possibility is that cortical control of non-lemniscal areas depends on the stimuli used and the specific experimental task and that the conditions used in the animal experiments so far do not elicit top-down control of SSA.

The thalamic reticular nucleus (TRN) is a laminar GABAergic nucleus that covers large parts of the thalamus and serves as interface to the cerebral cortex (Ohara and Lieberman, 1985; Pinault, 2004). TRN neurons show even stronger SSA than nuclei of the auditory sensory pathway, with SSA indices that double those of the (non-lemniscal) MGB (Yu et al., 2009). Moreover, TRN deactivation has been shown to affect the responses on MGB after (not during) the presentation of a deviant (Yu et al., 2009). This suggests that the deactivation does not influence the prediction error component of MGB responses, but potentially rather the encoding of the generative model of the sensory world. However, Yu et al. measured the effect of TRN deactivation in just one MGB neuron so these results should be interpreted with caution until replications are available.

In summary, there are only very few studies investigating corticofugal influences on presumed prediction error responses in IC and MGB. Only two studies show that SSA in IC and MGB is driven by top-down control (Yu et al., 2009; Bauerle et al., 2011).

## 6. Favoring Evidence for the Global Model Based on Manipulation of High-Order Expectations

An alternative approach to study the computational principles of the subcortical sensory pathway nuclei is to measure adaptation in subcortical sensory nuclei while manipulating predictions that are unlikely to stem directly from subcortical processing. Such predictions can be derived either from complex statistical regularities that are unlikely to be encoded in subcortical sensory structures or from cognitive representations that are characterized by high levels of abstraction.

One first step toward such an approach is to use paradigms that tap into so-called *meta-adaptation*. Meta-adaptation is a phenomenon where adaptation dynamics themselves adapt depending on changes in the context in which the adaptation dynamics occur (Robinson et al., 2016): Robinson and colleagues exposed Guinea pigs to repeated switches between quiet and loud environments. They observed that the adjustment in the dynamic range of neurons in IC accelerated after repeated exposure to the two different environments. Thus, the adaptation of the dynamic range adapted to the novel but familiar environmental context. This meta-adaptation effect largely attenuated after the experimenters deactivated auditory cortex using a cryoloop. Under the light of the PPF, the faster adaptation dynamics would result from the prediction that switches occur often. The result that meta-adaptation on IC depends on the integrity of the cerebral cortex can thus be interpreted as evidence that the generative model is encoded in auditory cortex, favoring the global model.

Malmierca et al. (2019) used an elegant paradigm with complex statistical regularities to investigate responses in the anesthetized rat's IC. The authors used as predictable entity a pattern of two tones that was presented in a repetitive manner (i.e., A-B-A-B-A-B…). To elicit prediction error, the pattern was rarely violated by a repetition of one of the tones (…A-B-A-B-B). The rationale was that the representation of the tone dyad A-B is putatively encoded at higher processing levels than the representation of a single tone typically exploited in SSA experiments. Neurons encoding prediction error in IC would therefore only respond to violations of the pattern if predictions encoded in higher levels are used to predict sensory input in the IC. The authors reported that only 14 of 281 measured samples of IC neurons, located in both lemniscal and non-lemniscal subdivisions of the nucleus, showed statistically significant prediction errors to violation of the patterns. The study was the first to investigate SSA in the subcortical sensory pathway with a paradigm that it likely represented in complex generative models in the brain. However, since the fraction of neurons with significant prediction error reported in the study (14/281≃4.98%) was close to the false-discovery rate of the study (α = 0.05), replications would need to confirm this effect unequivocally.

Yu et al. (2009) used a different approach to control predictability: They used a light to cue the onset of the auditory stimuli while recording from neurons of the anesthetized rat's MGB. They found that the visual cue resulted in significantly suppressed responses in 20 of 118 (≃17%) measured neurons and significantly enhanced responses in 23 of them (≃19.5%), both way above the false discovery rate of the study. Assuming that the causal link connecting the visual cue to the expectations on the auditory input is computed at a processing stage other than the MGB, we interpret these results as evidence for the global model. Favoring this interpretation, the authors show that deactivation of the TRN suppresses the effects of cuing in both directions; however, this result is once again shown in a single neuron and should be interpreted with caution until replications are available.

Lau et al. (2017) showed entrainment enhancement of the FFR in humans driven by high-order predictability using pitch contours of Mandarin syllables. The authors presented a target syllable in three different contexts: an unpredictable context, where the likelihood of the target was 1/3; a repetitive context, where all stimuli were repetitions of the target; and a patterned context, where the target was presented in a pattern of three syllables that was repeated over and over. The results demonstrated that the FFR entrainment was enhanced by predictability (i.e., that the FFR was more correlated to the stimulus waveform in the two predictable contexts than in the unpredictable context). In addition, the entrainment was stronger for the high-order predictability (i.e., in the patterned context) than when predictability was dictated by repetition. Although *predictability enhancement* cannot be interpreted as prediction error dynamics within the PPF, the result that predictability stemming from a higher level of abstraction has a stronger weight than predictability stemming from repetition in the strength of the FFR supports the global model.

**Figure 2:**
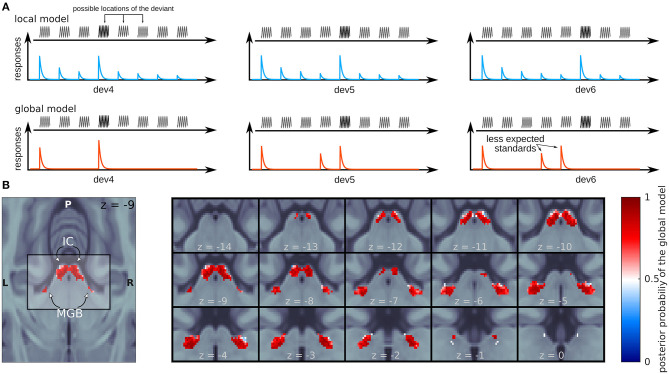
Evidence for the global model in the human IC and MGB. **(A)** Schematic view of the expected responses for the different trials by the local (blue) and the global (red) models. **(B)** Posterior probability map of the global (red) and local (blue) models. *z*-coordinates correspond to the MNI space.

The most recent evidence for the global model comes from a study in humans from our lab (Tabas et al., 2020) where we manipulated high-order predictions while preserving local stimulus statistics. We used fMRI to measure responses in the IC and MGB to a variation of the classical oddball sequence where the predictability of the deviants was manipulated using abstract rules. We disclosed to the participants that in each oddball sequence one of the standards at positions 4, 5, or 6 will be substituted by a deviant (Figure 2A). Since each position was equally likely across the experiment, after 3 repetitions of the standard subjects expect a deviant in position 4 with a likelihood of *p* = 1/3, after hearing 4 standards they expect a deviant in position 5 with *p* = 1/2, and after 5 standards subjects fully expect a deviant in position 6. According to the local model of the PPF, only the ratio between deviants and standards will have an effect on the strength of the responses to the deviant tones (Figure 2A, blue); according to the global model of the PPF, the responses will be the weaker the higher the likelihood of the tone according to the abstract rules (Figure 2A, red).

Using Bayesian Model comparison, we showed that responses in the IC and MGB (Figure 2B) were far more likely to be produced by a mechanism following the principles of the global model (where the magnitude of the response decreased with predictability) than by a mechanism following the principles of the local model (where the magnitude of the responses decreased with the number of times the stimulus has been repeated before). The global model was similarly prominent in both lemniscal and non-lemniscal sections of the MGB, revealing once again no particular functional organization of the human auditory pathway in respect of the PPF.

## 7. Subcortical Predictive Processing in Other Sensory Modalities

Although here we have focused on the auditory modality, it is likely that the processing architecture of other sensory modalities follows similar principles. Indeed, analogous functional and anatomical organizations have been found between the auditory and visual (Rauschecker, 2015), and visual and somatosensory (Pack and Bensmaia, 2015) systems. Moreover, if the auditory pathway is organized according to a global PPF, this organization should necessarily extend to other sensory modalities: otherwise the predictive potential of the global model would be largely under-exploited. There is indeed plenty of evidence that information across modalities is integrated and applied to sensory coding according to the principles of the PPF (see reviews, von Kriegstein, 2012; van Wassenhove, 2013; Talsma, 2015). In this section, we describe a few examples of predictive processing in the visual and somatosensory subcortical pathways.

Predictive coding was originally enunciated as a visual theory (Rao and Ballard, 1999). Most literature on visual predictive processing is concerned with the problem of extra-classical receptive field properties in response to concurrent stimulation (e.g., Aitchison and Lengyel, 2017). Some studies have, however, also considered how predictions on future events are used during the encoding of visual information in the subcortical visual pathway. Evidence for predictive processing of this kind has been reported in the retina (Hosoya et al., 2005; Kastner and Baccus, 2013; Howlett et al., 2017; Johnston et al., 2019; Kastner et al., 2019), including a study demonstrating SSA to movement in retinal bipolar cells (Ölveczky et al., 2007); in the superior colliculus, in the form of SSA to Gabor patterns (Jin and Glickfeld, 2020) and luminance (Boehnke et al., 2011); and in visual thalamus to location and polarity of light bars (Dhruv and Carandini, 2014). In the visual thalamus, predictive feedback has been suggested to stem from corticofugal efferents from primary visual cortex (Jehee and Ballard, 2009; Zabbah et al., 2014), but has yet not been demonstrated empirically. Thus, evidence to-date in the visual subcortical pathway is compatible with both, the global and local models of the PPF.

Adaptation to local stimulus statistics has also been reported in the mammal (Khatri et al., 2004; Mohar et al., 2013; Liu et al., 2017) and human somatosensory thalamus (Allen et al., 2016), but results are compatible with both, the local and global models of the PPF. One of these studies (Mohar et al., 2013) found a functional subdivision of the somatosensory thalamus similar to that of the animal literature in the auditory modality: non-lemniscal subdivisions showed stronger adaptation dynamics than their lemniscal counterparts. Evidence for the global model was provided by a study (Pais-Vieira et al., 2013) that considered the effect of anticipation on somatosensory thalamus during the activation of the facial whiskers of the rat. Pais-Vieira et al. found that effects of anticipation clearly present in somatosensory thalamus vanished after deactivation of the somatosensory cortex with muscimol. Perhaps the most compelling demonstration that the somatosensory pathway is organized according to the PPF is the common placebo effect, described by the PPF as a drastic reduction of pain sensation by the imposition of a strong analgesic prior (Büchel et al., 2014). Favoring the global model, reduction of activity to painful stimulation after the administration of a placebo has been found in the medulla (Matre et al., 2006; Eippert et al., 2009).

## 8. Conclusion

Converging evidence indicates that hierarchical predictive processing is a key feature, if not the principal encoding strategy, of subcortical sensory pathways. In the auditory modality, it is clear that encoding in the IC and MGB are strongly driven by expectations on the incoming stimuli. There is, however, still mixed evidence about the underlying mechanism of these expectations. We have summarized the two extreme possible architectures of the predictive processing network in two opposing views: a local model, where each stage in the hierarchy encodes its own representation of the stimulus and performs predictions on the representation of the immediately lower stage; and the global model, where a global prediction, encoded at higher processing stages, propagates downwards generating local predictions at all subsequent cortical and subcortical stages. In our review of the literature we have found a few studies favoring the local model, several studies favoring the global model, and a large number of studies whose results are agnostic to the architecture of the predictive processing network.

One possibility is that the feedback propagation of the global model is adapted according to the specific context. Electrophysiological experiments in animals are typically performed under anesthesia. This work has impressively shown that SSA is a fundamental automatic reaction of sensory systems rather than a phenomenon triggered by particular cognitive actions or arousal. In anesthesia, however, animals experience sounds under the same context: that of drug-induced coma. The two studies that investigated pure tone SSA on awake animals (Duque and Malmierca, 2015; Parras et al., 2017) demonstrated that SSA is pervasive in alert states, but used passive listening conditions. Whether the cognitive context and behavioral relevance of the stimuli might have deeper repercussions when more complex models of the sensory world are necessary in order to compute expectations has not being investigated yet. This possibility could explain why evidence for the global model in the IC of anesthesized rats was inconclusive (Malmierca et al., 2019), while there was strong evidence for the global model in awake human participants (Tabas et al., 2020). It is also possible that not all processing stages conform under the same context to the same model: different stages of the processing hierarchy might depend to a greater or smaller degree on high-level expectations. Differences between the two studies could, however, also be explained by the many methodological differences between animal and human studies and by potential species-specific differences in rodent and human sensory systems.

An important open question is exactly where the neural units that encode the model of the sensory world used to compute prediction error in the subcortical nuclei are located. Although in theory there is no reason why prediction error units could not encode the generative model in a multiplexed code, prediction error and the model are usually argued to be encoded in distinct populations (e.g., Friston, 2003, 2005; Bastos et al., 2012; Spratling, 2017; Keller and Mrsic-Flogel, 2018). However, in comparison with the evidence for the ubiquity of prediction error units, evidence for the existence of the generative model units is scarce in the cerebral cortex (Bell et al., 2016; Fiser et al., 2016; Walsh et al., 2020), and practically non-existent in subcortical areas. There is weak evidence that TRN neurons might have an active role on applying these models in MGB (Yu et al., 2009), but the fact that TRN neurons themselves show SSA render this hypothesis unlikely. According to the existing formulations of the PPF (Kiebel et al., 2008; Spratling, 2017; Keller and Mrsic-Flogel, 2018), each representational level should have a corresponding local model (see Figure 1B). This means that, if we accept that the IC and MGB encode different stages of the processing hierarchy, the MGB should have a local population of neurons encoding predictions that are transferred to the IC. Some algorithms actually locate the predictive model at the same processing stage, meaning that the populations encoding the model would actually reside in the IC (Spratling, 2017). There is, however, still no evidence for the encoding of these models in subcortical stages. Direct corticofugal connections exist all the way down to the cochlear nucleus (Winer, 2005a), so it is theoretically possible that all subcortical nuclei are located at the same hierarchical stage with respect to the PPF and that their corresponding model is located in primary auditory cortex. However, the presence of thalamo-collicular, thalamo-cochlear, and colliculo-cochlear efferents (Schofield, 2011) indicate that predictions are most likely also conveyed across different subcortical stages.

Another key ingredient necessary to understand the architecture of the PPF is the exact mechanism underlying the computation of prediction error and generation of predictions at each stage. Some PPF algorithms have suggested that prediction error might be computed by subtracting the predictions from the sensory input via inhibition (Wacongne et al., 2012). However, predictability leads to behavioral benefits and wrong predictions can sometime bias perception toward incorrect percepts (de Lange et al., 2018); an inhibitory account of the computation of prediction error would not be able to account for any of these effects. Moreover, the dependence of the repetition entrainment enhancement of the FFR on predictability and the enhancement of the responses in the MGB by visual cues (Yu et al., 2009) seem to indicate that predictions can enhance the sensory representation, rather than inhibiting it. Future models of the PPF face the challenge of reconciling these findings with the repetition/predictability suppression characteristic of prediction error.

Understanding the neural mechanisms underlying sensory processing is the only robust approach to understand perception. If, as enunciated by the global PPF model, sensory processing is a process of inference, we should remove all claims of perceptual objectivity, pay close attention to our priors and our internal models of the world, and question ourselves about the realities we cannot perceive just because they are not part of our model space. Although absolute inference is an unlikely scenario, since we must have formed our current models based on empirical experiences, it is possible that our reliance on inference grows more and more as we age (Lucas et al., 2014; Sherratt and Morand-Ferron, 2018; Cohen et al., 2020). SSA and the discovery of the encoding of prediction in subcortical sensory pathways have opened the gates to a deep exploration on sensory organization that might have strong philosophical repercussions on the way we understand what we call reality. If future work departs from paradigms that are unspecific about the underlying model of the sensory world, research on the PPF could lead us to the roots of the mechanisms that make us see, hear, and feel.

## Author Contributions

AT and KK reviewed the literature and wrote the manuscript. Both authors contributed to the article and approved the submitted version.

## Conflict of Interest

The authors declare that the research was conducted in the absence of any commercial or financial relationships that could be construed as a potential conflict of interest.
